# The Location Monitoring of Fatigue Crack Damage by Using the Spectral Area Extracted from FBG Spectra

**DOI:** 10.3390/s20082375

**Published:** 2020-04-22

**Authors:** Yan Zhao, DianYin Hu, Meng Zhang, Wei Dai, Weifang Zhang

**Affiliations:** 1 Research Institute of Aero-engine, Beihang University, 37 Xueyuan Rd., Haidian Dist., Beijing 100191, China; zy_buaa@buaa.edu.cn; 2 School of Energy and Power Engineering, Beihang University, 37 Xueyuan Rd., Haidian Dist., Beijing 100191, China; hdy@buaa.edu.cn; 3 School of Reliability and Systems Engineering, Beihang University, 37 Xueyuan Rd., Haidian Dist., Beijing 100191, China; zhangmeng123@buaa.edu.cn (M.Z.); dw@buaa.edu.cn (W.D.)

**Keywords:** spectral area, FBG sensor, crack location detection, structural health monitoring

## Abstract

In this paper, a new damage feature, spectral area, was extracted to effectively detect crack location by studying the deformation mechanism of fiber Bragg grating (FBG) reflection spectra. In order to verify the robustness and reliability of spectral area to detect crack location, the following work was carried out: Firstly, the strain information was extracted by extended finite element method (XFEM) with fatigue crack propagation. The transmission matrix method (TMM) was used to simulate FBG reflection spectra using numerical results. Secondly, the fatigue crack growth monitoring experiment based on FBG sensors was carried out, and the digital image correlation (DIC) method was used to measure the strain values at the placement of FBG sensors with crack propagation. The temperature characteristic test of FBG was carried out to investigate the influence of temperature variation on the spectral area. The results presented that the spectral area was insensitive to temperature variation and experimental noise, and was greatly sensitive to the complex non-uniform strain field cause by crack damage. Moreover, compared with the 5 mm FBG sensor, the 10 mm FBG sensor showed a larger critical detection range for crack damage. Therefore, the spectral area can be used as a reliable damage feature to detect the crack location quantitatively based on the simulated and experimental results.

## 1. Introduction

Fiber Bragg Grating (FBG) sensor has become one of the most promising sensors in the field of structural health monitoring (SHM) [1,2]. This is because it has many advantages [3], such as light weight, small size, corrosion resistance, electromagnetic interference resistance, etc. Fatigue crack damage often occurs around holes in aeronautical structures, which causes the stress/strain field variation of structures with crack propagation [4]. It is difficult for global sensors to monitor effectively the local small-scale damage of key structures [5]. However, the strain field variation at structural critical positions can be sensed sensitively by FBG sensors as a local-sensor, which shows great potential in crack damage monitoring by analyzing the deformation of reflection spectra in the process of fatigue crack propagation. In fact, the FBG reflection spectrum can be predicted theoretically with known strain information and FBG structural parameters using the transmission matrix method (TMM) [6]. On the contrary, it is very difficult to detect the location of crack damage in the case of unknown strain states and known deformation spectra.

Many studies have shown that the FBG reflection spectrum was distorted due to the inhomogeneous strain field caused by damage [7]. Many damage features factors were extracted from the distorted spectra based on the response. Zhang, W. et al. [8] presented that the central wavelength of FBG reflection spectra shifts with the variation of structural strain field. When the axial uniform strain sensed by FBG sensors increases, the central wavelength increases linearly; while the spectra distorted by axial non-uniform strain, the central wavelength shift does not follow the linear relationship. Simultaneously, the temperature changes can also cause the central wavelength shift [9]. However, the structural strain field is also constantly changing without crack damage under the external loadings. Moreover, considering that the aircraft structure design usually follows the damage tolerance theory [10]. Only when the crack grows to a critical length, the structure will fail. Thus, more attention should be paid to the crack propagation stage than to the crack initiation stage [11]. What is more, the damage features are more susceptible to crack damage and obtuse to other external conditions to monitor structural crack damage. Thus, the center wavelength is not suitable for crack location detection. Liu Q et al. [12] found that the bandwidth broadening of the FBG reflection spectra was caused by the strain gradient. Bao P. et al. [13] analyzed the singularity of spectral signals by using the spectral cross-correlation analysis algorithm and cross-correlation function sequence to determine crack initiation and propagation. Mizutani, Y. et al. [14] extracted the full width at half maximum (FWHM) form FBG reflection spectra to evaluate the strain field caused cantilever beam test, which showed FWHM is sensitive to non-uniform strain. Hu, H. et al. [15] found the high correlation between the width at a quarter of peak ($w_{1/4}$) form distortion spectrum and crack density. The result showed the $w_{1/4}$ is an effective indicator for monitoring transverse crack density. Takeda, S. et al. [16] monitored the debonding of composite repair patches by using FBG sensors, which demonstrate the effectiveness of intensity ratio extracted form FBG reflection spectra as a damage index. Rito, R. L. [17] found that shape variations of FBG reflection spectra can be used to study the crack growth. However, how to monitor and locate structural cracks is always a hot issue in the state of unknown structural strain field.

In this paper, a new damage feature, spectral area, is extracted from the FBG distortion spectra acquired by fatigue crack propagation experiment and simulation of reflection spectra, which is proposed firstly to monitor location of crack damage. The spectral area represents the reflection ability of an FBG in a certain wavelength range. In order to study the performances of spectral area in detecting crack position, the following work was carried out. Firstly, in order to verify the generality of spectral area, two kinds of FBG sensors with different grating lengths (the grating length is 5 mm and 10 mm) are used to monitor and locate the crack damage. Secondly, the strain information was obtained by using extended finite element method (XFEM) with crack propagation, which is used as the input for spectral simulation. The TMM is used to simulate the FBG reflection spectra. The calculation method of spectral area was given. The spectral area extracted from the simulated spectra is used to study location detection of the crack damage. The fatigue crack propagation can be effectively simulated by XFEM, which need not be re-meshed near the crack tip [18]. Thirdly, the fatigue crack propagation experiments based on the crack damage monitoring were carried out by using the FBG sensors glued to the specimens back. Simultaneously, the strain information at the FBG sensors placement position was measured in situ using the digital image correlation (DIC) method [19]. The DIC, a representative full-field non-contact optical method, is used to measure the surface deformation of structures under various loads [20], which overcomes the disadvantage that traditional resistance strain gauges can only obtain the average strain along the grating direction. The results of DIC processing and numerical simulation are used to quantitatively analyze the deformation mechanism of spectral area. Then, the temperature response experiment of FBG sensors was carried out to study the effect of temperature on the spectral area. The response behaviors of the FBG reflection spectra and spectral area are analyzed in detail at different temperatures. Then, the experimental and numerical results show that there is a significant inflection point in spectral area curves with crack propagation. The location detection of fatigue crack damage can be performed by identifying the location of the inflection point. Finally, the detection performance of FBG sensors with the grating lengths of 5 mm and 10 mm is studied.

Compared with previous studies, the proposed method has five main advantages. Firstly, the deformation mechanism of spectral area caused by crack damage and temperature variation is revealed quantitatively by theoretical analysis, spectral simulation and experimental verification. Next, a new damage feature, spectral area, is proposed and verified to effectively detect the crack location. Then, the location detection of crack damage can be performed by identifying the inflection point of spectral area curve. Then, the robustness of spectral area to experimental noises and temperature changes is verified by carrying out fatigue crack monitoring experiments and temperature feature experiments. Finally, the detection performance of two FBG sensors with different grating lengths is studied. Compared with the 5 mm FBG sensor, the 10 mm FBG sensor shows a larger critical detection range to crack damage.

## 2. Theoretical Analysis 

### 2.1. The Analysis of FBG Strain and Temperture Feature 

The FBG is an optical device with periodic perturbation of the refractive index along the grating length of the fiber, which developed by the core being exposed to a strong light interference pattern. Moreover, the grating period and effective refractive index of FBG will change if the variations of temperature and strain were sensed by FBG. For the single-mode FBG, its peak wavelength $\lambda_{B}$ can be expressed as [21]:

(1) $$\lambda_{B}=2n_{eff}\Lambda$$

where the $n_{eff}$ is effective refraction index of fiber core and the Λ is the grating period.

According to previous research [22], if the FBG is only affected by the temperature field change, the shift of the central wavelength of the FBG can be expressed as:

(2) $$\frac{\Delta \lambda_{B}}{\lambda_{B}}=\left(\xi +\alpha +\frac{1}{n_{eff}}K_{wg}\alpha \right)\Delta T$$

(3) $$\xi =\frac{\partial n_{eff}}{\partial Tn_{eff}}$$

(4) $$\alpha =\frac{\partial \Lambda }{\partial T\Lambda }$$

(5) $$K_{wg}=\left(\frac{{\left(\Delta n_{eff}\right)}_{ep}}{\partial \Lambda }+\frac{\partial n_{eff}}{\partial a\partial \Lambda }\Delta a\right)\Lambda$$

where the ΔT is the temperature variation, the ξ is the thermal optical coefficient of fiber grating, the α is the thermal expansion coefficient of the fiber grating, the ${\left(\Delta n_{eff}\right)}_{ep}$ is the elastic optical effect caused by the thermal expansion, and the $\frac{\partial n_{eff}}{\partial a\partial \Lambda }$ is the waveguide effect caused by the variation of the fiber core diameter due to the thermal expansion. The $K_{wg}$ is the wavelength drift coefficient of the fiber grating caused by the waveguide effect. The ξ and α are constants for a material, and the period and effective refractive index of FBG are linear with temperature [23]. Therefore, the temperature change only makes the central wavelength drift, and has little effect on the deformation of FBG reflection spectrum shape.

When the uniform strain was sensed by FBG sensor, the relationship between the central wavelength moving and axial uniform strain is linear, and the spectral shape hardly changes. When the inhomogeneous strain was sensed by FBG sensor, the central wavelength of FBG was shifting, but the spectral shape was also distorted. It can be seen from Section 2.2 that when the crack tip approaches the FBG sensor, it mainly senses the non-uniform strain. In order to study the response of FBG reflection spectrum to uniform strain and non-uniform strain, Peters et al. [24] firstly proposed the TMM method to simulate the deformation spectra of FBG under non-uniform strain. When the non-uniform strain is sensed axially by FBG sensor, it can be regarded as N fiber gratings with uniform strain. The axial strain $\varepsilon_{z}\left(y_{i}\right)$ of any fiber grating segment changes the period $\Lambda_{i}$ and effective refractive index ${\left(\Delta n_{eff}\right)}_{i}$:

(6) $$\Delta \Lambda_{i}=\Lambda_{i}\varepsilon_{y}\left(y_{i}\right)$$

(7) $${\left(\Delta n_{eff}\right)}_{i}=-\frac{n_{eff}^{3}}{2}\left[\left(1-\nu \right)p_{12}-\nu p_{11}\right]\;\varepsilon_{y}\left(y_{i}\right)$$

where the $\Delta \Lambda_{i}$ is period variation. In Equation (7), the ${\left(\Delta n_{eff}\right)}_{i}$ is variation of effective refractive index, ν is Poisson’s ratio, and $p_{11}$ and $p_{12}$ are the Photo-elastic coefficient. The inhomogeneous strain can lead to the spectra broadening and the decrease of spectra reflectivity.

### 2.2. The Strain Calculation and Reflection Spectrum Simulation

**Figure 1:**
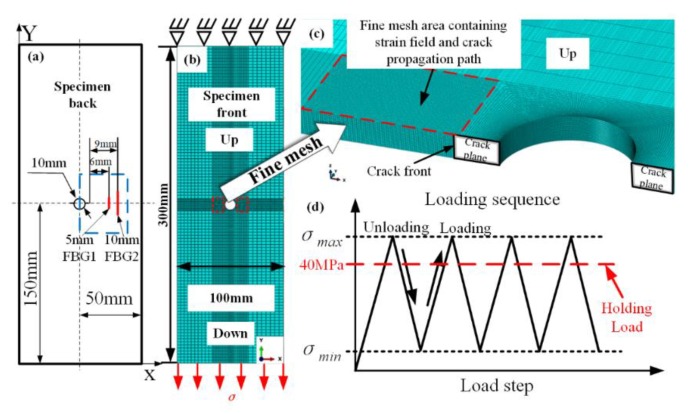
(**a**) The dimensions of the specimen; (**b**) the finite element model of specimen; (**c**) the fine mesh area containing strain field and crack propagation path; (**d**) cyclic loading sequence.

The XFEM was used to simulate the fatigue crack propagation, which can better simulate a complex unknown field with crack growth than the traditional finite element method. Aviation aluminum alloy 7075-T6 is used in the fatigue crack damage monitoring experiments, and the main mechanical property parameters of specimen are shown in Table 1. The dimensions of the specimen are 300 mm×100 mm×1 mm, with a center hole of 10 mm, as shown in Figure 1a. In order to promote the cracks initiation, 1 mm pre-cracks were introduced around the hole using electric-discharge machining (EMD).

First, the finite element model of specimen is created using ABAQUS software. The placement position of FBG sensors, the three-dimensional model of the specimen, and the fine mesh are shown in Figure 1. The boundary condition is that the top is fixed and the bottom is loaded, as shown in Figure 1b. In order to make the fatigue crack in the stable crack growth region, the stress intensity factor ($7.24\;\mathrm{MPa}\sqrt{m}$) and stress (50 MPa) for the specimen are calculated by NASGRO database (v8.01, Southwest Research Institute^®^ (SwRI), San Antonio, TX, USA). Simultaneously, the result of the NASGRO database is basically consistent with the stress intensity factors ($7.08\;\mathrm{MPa}\sqrt{m}$) calculated by FEM under 50 MPa loading. Thus, the load amplitude and the stress ratio were set to 50 MPa and 0.1, respectively. Bergara A et al. [25] used an 8-noded hexahedrons element with reduced integration (C3D8R) as the unit type to successfully simulate crack propagation in the complex structures front. The C3D8R is used for three-dimensional model, and fine mesh is used for the fatigue crack propagation path and strain field of crack tip, as shown in Figure 1c. The size of refined mesh is 0.04 mm×0.01 mm×0.1 mm. Furthermore, one workstation (Intel CPU 2.30 GHz X24 and 64 GB RAM) is used for numerical simulation. In order to ensure that the future experimental data is obtained under constant stress, and avoid crack retardation and crack arrest [26] behavior caused by high holding load, the holding load was set to 40 MPa. The cyclic loading sequence of the finite element simulation is shown in Figure 1d.

The two FBG sensors with different gating length (5 mm and 10 mm) are used for monitoring location of fatigue crack damage, and there the placement position is shown in Figure 1a. There are three purposes: (1) to prove the generality of the damage characteristic parameters for the location monitoring of metal fatigue cracks. (2) to investigate the robustness of the damage feature to experimental noise. (3) to contrast sensitivity of FBG sensors with different grating lengths for crack damage. Moreover, considering the damage tolerance theory, the placement position of FBG1 sensor (the length of grating is 5 mm) is about 6 mm away from the pre-crack tip. The FBG2 sensor (the length of grating is 10 mm) is placed about 9 mm in front of the pre-crack tip. This is to avoid the interference of two FBG sensors by the same plastic zone. The placement position of FBG3 is on the specimen-2 surface at the same position with FBG1. The mechanical properties of FBG1, FBG2 and FBG3 are shown in Table 2.

**Figure 2:**
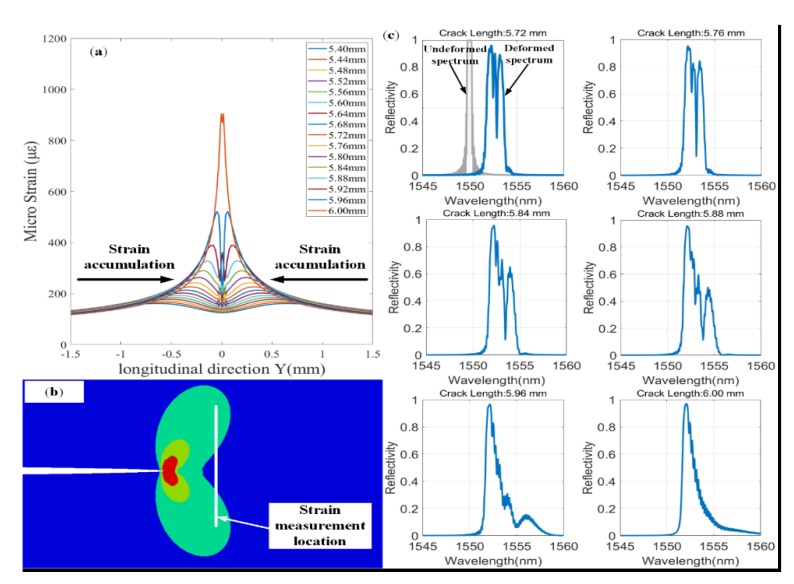
(**a**) The strain variation of FBG1 sensor placement position with crack propagation; (**b**) Schematic diagram of crack tip plastic zone and strain extraction location; (**c**) the simulated spectra at FBG1 placement position with the different crack length.

The longitudinal strain (E_yy_) information at the FBG1 and FBG2 placement positions is extracted by XFEM with fatigue crack propagation. Figure 2a shows the part of strain values of FBG1. As the crack tip approaching the FBG sensor, the inhomogeneous strain field is sensed by the FBG. The gradient of the non-uniform strain field also increases gradually along crack propagation direction, as shown in Figure 2a. Figure 2b shows Schematic diagram of crack tip plastic zone and strain extraction location, which explains why the strain value increased first and then decreased with crack propagation. Furthermore, the FBG sensors are generally used in small ranges of crack damage monitoring, this is because the non-uniform strain area near the crack tip is relatively small.

In order to clearly show the distortion behavior of FBG reflection spectra, the reflection spectra of FBG1 is simulated by TMM. Figure 2c shows the results of FBG1 reflection spectra by using the stain information obtained from FEM. The detailed parameters of FBG1 are to see Table 2.

The undeformed reflection spectrum of FBG1 is simulated to compare with its deformed spectrum, as shown in Figure 2c. The central wavelength of the undeformed reflection spectrum is 1550 nm. With the crack growth, the reflection spectrum shifts to the long wavelength direction, and spectral distortion occurs, including spectral broadening and multi-peak appearance. Moreover, the non-uniform strain field also leads to the reduction of peak reflected power. Therefore, this phenomenon is consistent with the above theoretical analysis, which also leads to the change of spectral area. The quantitative study of spectral area is given in Section 2.3.

### 2.3. Calculation Method of Damage Feature-Spectral Area

The spectral area of reflection spectrum is proposed as a feature parameter of FBG. It can quantitatively monitor the crack position by analyzing the variation trend of spectral area with crack propagation. As mentioned in Section 2.2, when the FBG is attached to the specimen surface and near the crack tip, the reflection spectra will be distorted, and the spectral area will be changed. 

**Figure 3:**
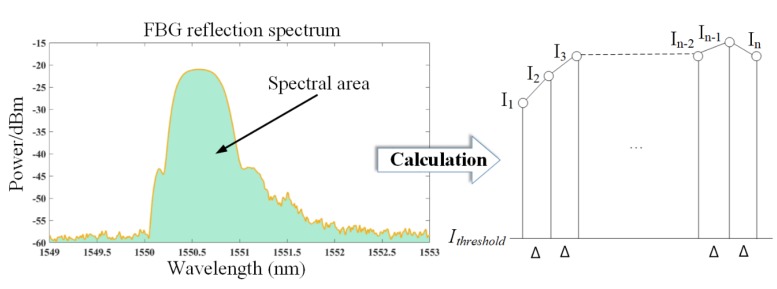
The schematic diagram of spectral area calculation method.

Figure 3 shows the schematic diagram of the spectral area calculation method. In fact, the reflection spectrum acquired by the interrogator consists of many discrete points. These discrete points can be defined as $I_{1},\;I_{2},\;I_{3},\ldots ,I_{n}$, and the sampling interval is Δ.

The region surrounded by the primary peak of the reflection spectrum with reflectivity higher than a certain threshold ($I_{threshold}$) is taken as the spectral area, which is equal to the sum of trapezoid areas composed of all adjacent sampling points and the threshold ($I_{threshold}$). The area can be calculated by Equation (8).

(8) $$S=\frac{\left(I_{1}-\left(I_{\mathrm{threshold}\;}\right)\right)+\left(I_{2}-\left(I_{threshold\;}\right)\right)\Delta }{2}+\ldots +\frac{\left(I_{n-1}-\left(I_{threshold\;}\right)\right)-\left(I_{n}-\left(I_{threshold\;}\right)\right)\Delta }{2}\;=\frac{\Delta }{2}\left(2I_{threshold}\left(n-1\right)+I_{1}+2I_{2}+2I_{3}+\ldots +2I_{n-1}+I_{n}\right)$$

where the n is the number of sampling points of the main peak of the reflection spectra of each FBG, the Δ is the sampling interval of the FBG interrogator, and $I_{i}$ is the reflectivity corresponding to the $i_{th}$ sampling point. The $I_{threshold}$ is the threshold of reflection spectrum. The preprocessing results of the experimental data show that the power of the FBG reflection spectra is above −60 dBm. To evaluate the area change behavior of the entire static spectrum, the $I_{threshold}$ is set to 0 for the simulated spectra and −60 dBm for experimental spectra.

**Figure 4:**
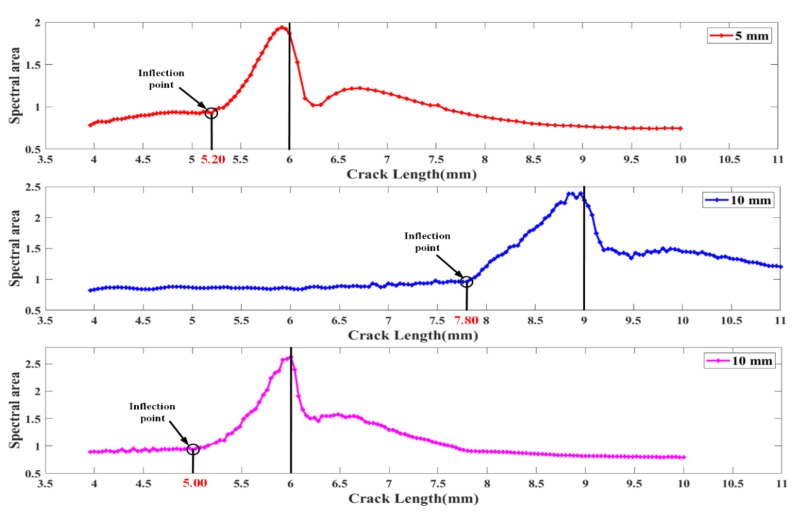
Calculated spectral area for simulated FBG sensors with different grating length responses under the strain extracted by XFEM.

The spectral area is calculated from the simulation results of the FBG reflection spectra (including FBG1, FBG2 and FBG3), as shown in Figure 4. When the crack tip is far away from the FBG sensors, these spectral area are almost unchanged, which shows that the external loadings and uniform strain have little effect on the spectral area. Then, the crack tip is near the FBG sensors, the inhomogeneous strain with a large gradient is sensed by FBG senses, and the reflection spectra are distorted. Moreover, the spectral area shows a monotonous and rapid growth trend while the crack tip distance to the FBG sensors is 0.8 mm, 1.2 mm and 1.00 mm, respectively. After the crack passes through the sensor, the reflection spectra and the spectral area tend to resume to their initial state. Because the unloading of the elastic strain material behind the crack tip. The crack tip position can be determined by identifying the obvious inflection point of the spectral area curves. Therefore, the damage feature, spectral area, measured from the simulated spectra has a good performance in the detection of crack locations. Furthermore, it is obvious from Figure 4 that the 10 mm FBG indicates a larger critical detection range than the 5 mm FBG. In Section 3, the performance of spectral area in fatigue crack growth experiment is discussed, and the robustness of spectral area to the experimental noise is evaluated by fatigue crack propagation experiment and temperature feature experiment.

## 3. The Experimental Procedure

### 3.1. The Fatigue Crack Growth Experiment Based on Crack Damage Monitoring 

**Figure 5:**
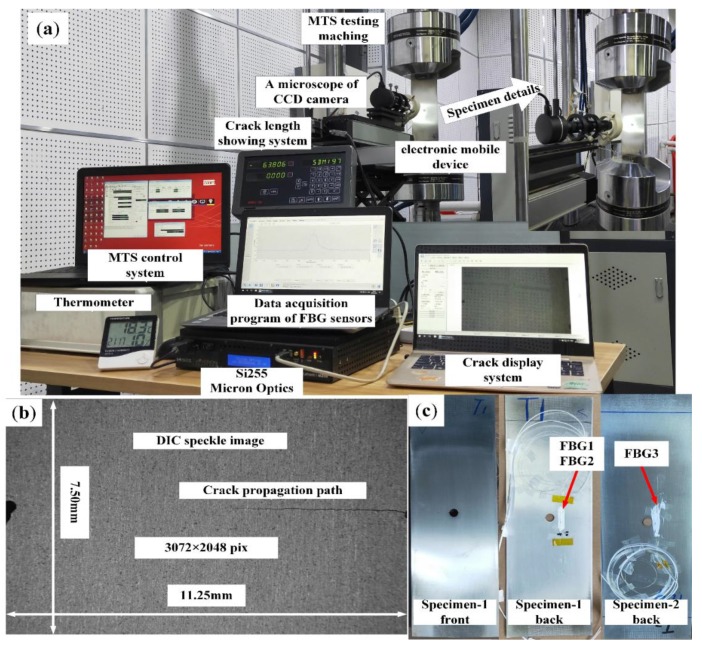
(**a**) The experiment platform for fatigue crack propagation and the specimen-1; (**b**) the DIC speckle image; and (**c**) the physical drawings of FBG1, FBG2 and FBG3 are on specimen-1 and specimen-2.

An experiment platform for fatigue crack damage monitoring based on FBG sensors and the DIC method was built, which included a fatigue cycle loading system, crack measurement system, DIC processing system, and an FBG sensors data acquisition system, as shown in Figure 5a. The top of the specimen is fixed, the bottom is loaded, and the constant amplitude loadings (the maximum tensile is 5 kN and stress ratio is 0.1) with a frequency of 10 Hz by the hydraulic MTS testing machine. The crack measurement system can display the crack propagation path and measure the crack length, as shown in Figure 5a. 

The DIC processing system includes specimen preparation, data acquisition and data analysis. The speckle was randomly sprayed on the front of the specimen, and the speckle image (about $11.25\times 7.50{\;\mathrm{mm}}^{2}$), as shown in Figure 5b. The purpose of specimen preparation is to track the deformation of digital image in reference images and current images. For data acquisition, a six-megapixel CCD camera was used to capture digital images for DIC analysis under different crack lengths. All the reference images and current images of DIC analysis were captured under 4 kN, which is 3072 × 2048 pixels and the spatial resolution of digital image is about 3.60 μm. The Ncorr was used for DIC analysis, which is an open-source software. Considering the calculation cost, the subset space and the strain subset radius are 6 and 15, respectively. 

Then, the FBG1 and FBG2 were bonded on the surface of specimen-1 with epoxy resin adhesive, and the specific pasting position is shown in Figure 1a. Figure 5c shows the physical drawings of FBG1, FBG2 and FBG3 on specimen-1 and specimen-2. The FBG1 was placed away from the crack tip to study the critical detection range of FBG to crack damage in specimen-1. The distance between FBG2 and FBG1 was 3 mm, to avoid being affected by the same plastic zone near the crack tip. Moreover, the FBG3 (grating length of 10 mm) was placed on the front of specimen-2, and its placement position is the same as that of FBG1 as a comparison sensor. The reflection spectra response of each FBG sensor was acquired by a Micron Optics si255 interrogator when capturing digital images. The acquisition resolution and wavelength range of interrogator were 0.008 nm and 160 nm, respectively. 

Furthermore, the test ambient temperature was kept constant (18 ± 1 °C) through central air-conditioning, and the effect of temperature changes on the FBG spectral area is not considered during the fatigue crack propagation experiment. The temperature characteristics of FBGs are studied in Section 3.2.

### 3.2. The Experiment for Temperature Properties of FBG Sensors 

**Figure 6:**
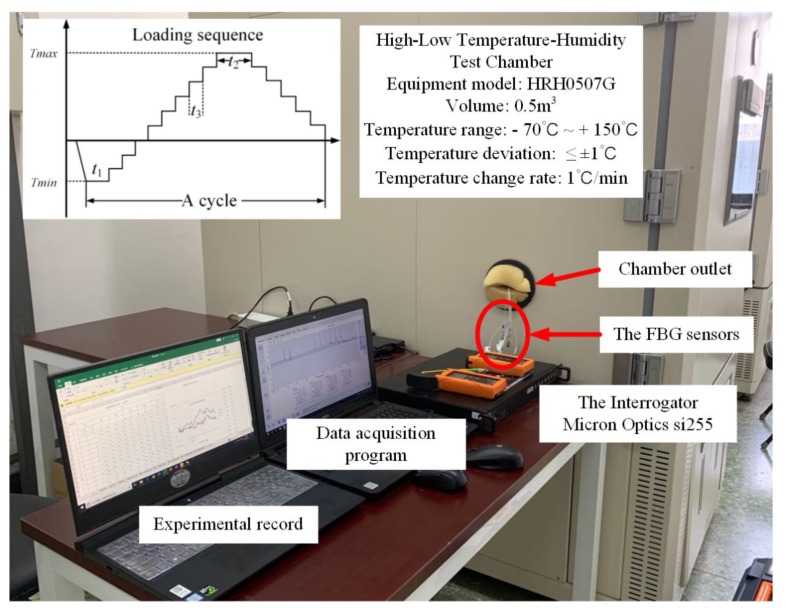
The temperature characteristics experimental platform of FBG sensors.

In order to investigate the temperature response of FBG spectral area, the temperature characteristics experiment platform was built, which included a High-Low Temperature-Humidity Test Chamber and a data acquisition system, as shown in Figure 6. The Test Chambers were produced by HongRuiYiQi, and the detailed parameters are shown in Figure 6. The wavelength range of the data acquisition system (Micron Optics si255) was 1460–1620 nm, and the resolution was 0.008 nm. The FBG sensor was connected to the Micron Optics si255 interrogator through the chamber outlet. In order to eliminate the influence of stress on the reflective spectra of the FBGs, the FBG sensors were pasted on the substrate whose thermal expansion coefficient is far greater than that of FBG material (the substrate plays a supporting role). When pasting the sensors, the FBG sensor was ensured to be straight and free from force, and then the base with the FBG sensor was put into the Test Chambers. These FBG sensors, which are the same as the fatigue crack monitoring test in Section 3.1, were used for the temperature characteristic test, and their main properties are shown in Table 3. As a contrast experiment, the grating length of FBG4 and FBG5 is 5 mm, and that of FBG6 and FBG7 is 10 mm. The temperature measurement range was −60 °C~ +110 °C, and experimental data of reflection spectra were acquired every 5 °C. The temperature gradient loading sequence is shown in Figure 6. The temperature holding time *t*_1_, *t*_2_ and *t*_3_ were 60 min, 60 min and 30 min, respectively. 

## 4. Experimental Results and Discussions 

### 4.1. The Strain Measured by DIC Method and Experimental Spectra

**Figure 7:**
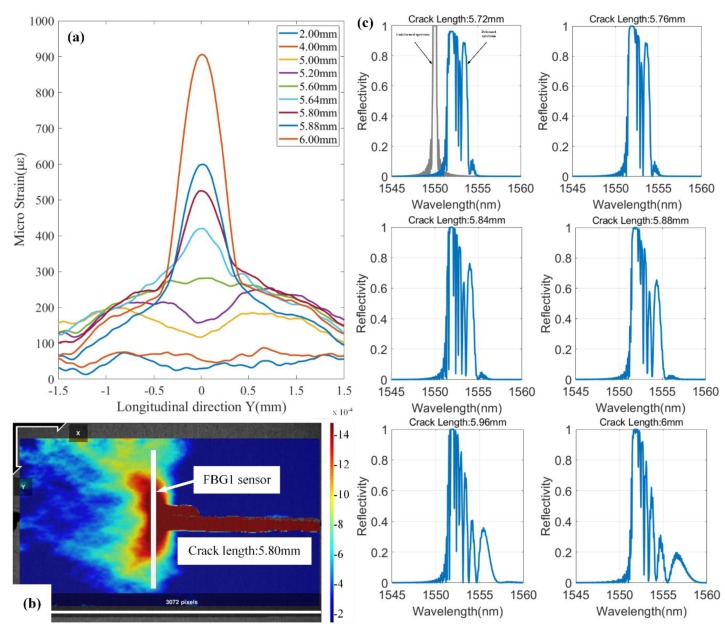
(**a**)The strain along the grating direction sensed by FBG1 sensor under different crack lengths; (**b**) The longitudinal strain field at the crack tip is measured using the DIC method; (**c**) Reflected spectra of FBG1 at different crack lengths.

The purpose of the fatigue crack growth experiment based on DIC method is to measure the strain information in the FBG sensors placement position. The principle of DIC method is to track the same subsets between two images captured the reference images (undeformed images) and current images (deformed images) [27]. The full-field strain measurement method overcomes the defect that the traditional strain gauge can only measure the average strain along grating. Figure 7a presents the strain distribution along the FBG1 grating under different crack lengths. At a crack length of 5.80 mm in Figure 7a, the strain distribution measured by DIC method is shown in Figure 7b. Under the condition of different crack length, the strain loadings along the grating reveals the stress concentration at the crack tip of specimen with a hole. Moreover, the “butterfly shape” at the crack tip is shown in the experimental results (Figure 7b), which accords with “Von Mises” criterion. Figure 7b gives the schematic diagram of the FBG1 placement position, which provides support for the analysis of the influence of longitudinal strain field on FBG. Furthermore, the strain loadings sensed by the FBG1 sensor were approximately uniform, while the crack tip was far from the FBG1. The non-uniform strain loadings with high strain gradient were gradually sensed by the FBG1 sensor with the crack propagation. Moreover, the strain measured using the DIC method in Figure 7a has approximate strain values to the numerical results with the same crack lengths in Figure 2a. Figure 7c shows that the reflected spectra were obtained through the fatigue crack propagation experiment.

The strain field sensed by FBG sensors changed continuously with the crack propagation. These reflection spectra were influenced by non-uniform strain field. In addition, these reflection spectra were inevitably disturbed by noise throughout the experiment. Therefore, these spectral areas extracted from the experimental spectra could help us study the robustness of spectral area to experimental noise. The reflection spectra of FBG1 are shown in Figure 7c. Compared with Figure 2c, they show a similar response to the simulation spectra using TMM. The original reflection spectrum presents a narrow-bandwidth Gaussian envelope—see Figure 7c. 

It can be seen from Figure 7 that when the crack approaches the FBG1 sensor, the strain components sensed by the FBG1 sensor change from uniform strain to non-uniform strain. This is because metal materials can easily form a plastic zone at the crack tip with crack propagation [4]. The FBG1 sensor was subject to strain loadings of plastic zone near the crack tip. Compared to the original spectrum, the reflection spectra have changed greatly as follows: (1) Distortion of reflection spectra. The primary peaks were divided into multiple peaks. (2) The spectra shifted towards the long wavelength direction while the crack tip approaching the FBG1 sensor. (3) The broadened bandwidths of reflection spectra were shown. The reflection spectra of FBG1, FBG2 and FBG3 were acquired in the whole crack growth process. Moreover, these experimental data were used to study the performances of spectral area for damage sensitivity, location monitoring of fatigue crack and noise robustness.

### 4.2. The Location Detection of Fatigue Crack Damage using Spectral Area

**Figure 8:**
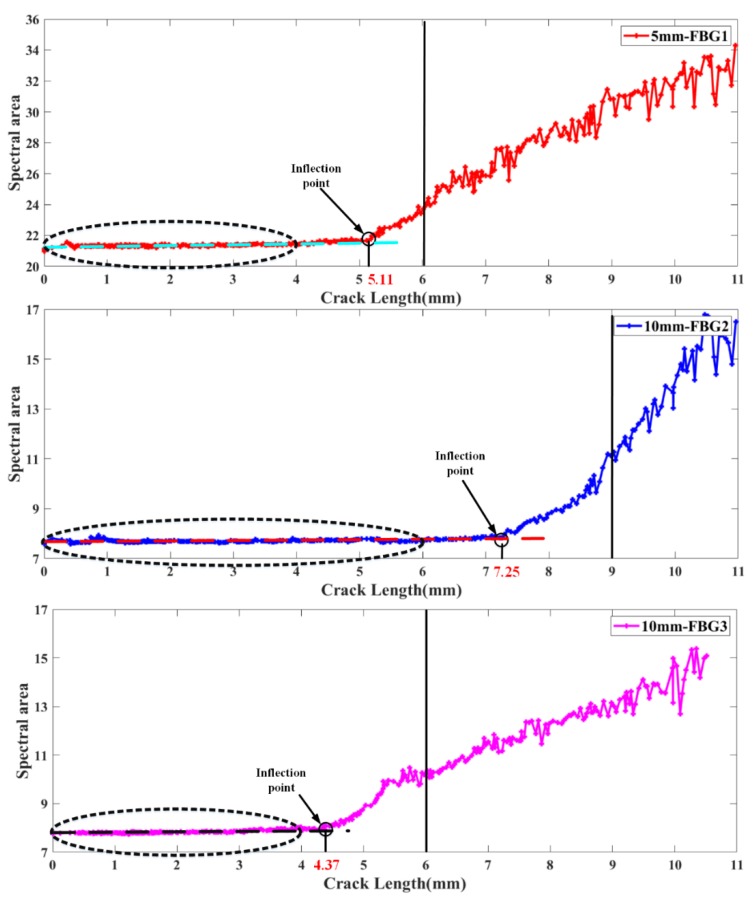
Experimental Damage feature spectral area variation with different crack lengths.

The spectral area of these reflection spectra obtained from fatigue crack growth experiments were calculated to detect the location of fatigue crack damage. The calculation results of the spectral areas of FBG1, FBG2 and FBG3 with crack propagation are shown in Figure 8. First, when the crack tip is far away from the FBG sensors, these spectral areas hardly change, which is consistent with the results calculated from the simulated spectra. This shows that the spectral area is hardly affected by uniform strain and external loading, because the strain gradients sensed by FBG sensors are not large enough. Then, the values of the spectral areas were fitted linearly with crack lengths of 1 mm–4 mm (for FBG1 and FBG3) or 1 mm–6 mm (for FBG2). The point was defined as the “inflection point”, which met the following two conditions: (1) near the fitting curve; (2) an obvious upward trend after the point. As the crack tip approaches the FBG sensor, the result of the spectral area shows a significant inflection point, as shown in Figure 8. A similar inflection point also appears in Figure 4. This shows that the non-uniform strain has a greater impact on the spectral area. After that, the spectral area monotonically increases. However, the simulated spectral area tends to decrease after the crack tip passes through the FBG sensors. This is mainly because the fiber grating is stretched due to the crack opening displacement during the experiment. Moreover, the distance from the inflection point to the FBG sensor placement position is defined as “critical detection range”. The critical detection ranges of FBG1, FBG2, and FBG3 are 0.89 mm, 1.75 mm, and 1.63 mm, respectively. Compared with FBG1, these spectral area of FBG2 and FBG3 present the larger critical range for crack damage. In addition, when the spectral area of FBG1 gradually increases, the spectral area of FBG2 does not change concurrently, which proves the robustness of the spectral area to experimental noise. Therefore, spectral area is insensitive to external load and uniform strain, sensitive to non-uniform strain and robust to noise. The spectral area can be used as a reliable and sensitive detection index for crack location under experimental complex strain fields and noise interference.

### 4.3. Study on Temperature Characteristics of FBG Sensors

**Figure 9:**
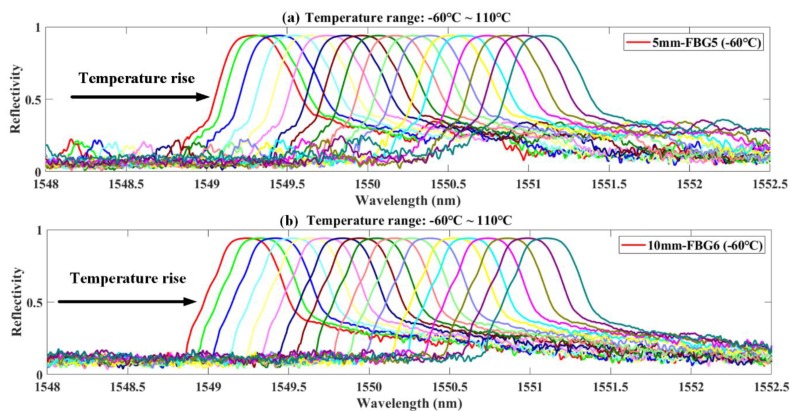
Reflection spectra of FBG5 and FBG6 under different temperatures.

The reflection spectra acquired from the temperature experiment are used to analyze the influence of temperature on the spectral area. The reflection spectra of some FBG sensors during the experiment are shown in Figure 9. The grating lengths of FBG5 and FBG6 are 5 mm and 10 mm, respectively. The data were collected every 5 °C in a temperature range from −60 °C to +110 °C. The experimental data of these sensors are affected by temperature loadings and noise interference. The original reflection spectra of the FBG sensors are narrow-band Gaussian envelopes. With the increase in temperature (from −60 to +110 °C), the reflection spectra shift towards long wavelength direction, and the reflection spectra were not distorted. There is no multi-peak phenomenon in the reflection spectra. The reflectivity and bandwidth of the reflection spectra have almost no variation.

**Figure 10:**
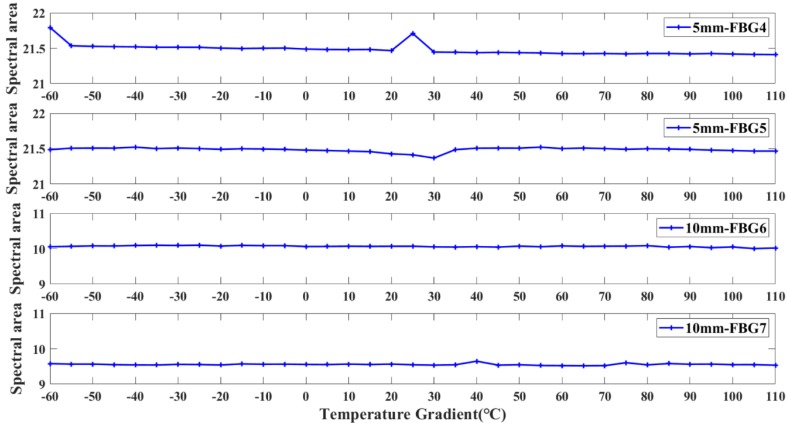
The variation of spectral area under different temperature.

Figure 10 shows the change behavior of the damage characteristics (spectral area) of the FBG sensors, with grating lengths of 5 mm and 10 mm and with temperature change. It is clear from Figure 10 that the spectral area of FBG has almost no change under the effect of temperature gradient. As the theoretical analysis in Section 2.1, the temperature variation can only make the central wavelength drift, and hardly change the spectral shape. Temperature variations have little effect on the spectral area of FBG sensors with grating lengths of 5 mm and 10 mm. The range of the spectral area (maximum value minus minimum value) is less than 0.4, which can be ignored. Therefore, the spectral area is an effective damage feature which is insensitive to temperature, external load and uniform strain, sensitive to non-uniform strain, and robust to noise.

## 5. Conclusions

In this work, the location detection of fatigue crack damage was studied using FBG sensors in aluminum alloy 7075-T6. A new and reliable damage feature, spectral area, was proposed to monitor the damage location. Firstly, the fatigue crack propagation was simulated by XFEM, and the strain information at the FBG sensors’ placement position was measured. The structural strain values obtained by XFEM were used as the input of the spectral simulation using TMM. The variation behaviors of the spectral areas extracted from the simulated reflection spectra were studied with crack propagation. Then, the fatigue crack growth experiment based on the crack damage monitoring was carried out using the FBG sensors, and the strain information of three FBG sensors was obtained using the DIC method. The deformation mechanism of the spectral area was analyzed by measuring the strain variation at the FBG placement with crack propagation. Compared with the results of the simulation and experiment, the distortion of spectral area has the same trend. The damage feature, spectral area, was extracted from experimental and numerical results to monitor the location of crack damage. Finally, the temperature characteristics experiment based on FBG sensors was carried out to study the influence of temperature on the reflection spectra and spectral area. The simulated and experimental results show that the spectral area can be used as a reliable and sensitive damage feature to detect the crack location. Through the above work, the main conclusions are as follows:

First, the deformation mechanism of spectral area is revealed quantitatively by theoretical analysis, spectral simulation and experimental verification. As the average strain sensed by FBG gradually increases, the spectrum shifts toward the long wavelength. As the strain gradient perceived by FBG gradually increased, the reflection spectrum is distorted, accompanied by spectral splitting, and the bandwidth became wider. What is more, the calculation method of spectral area is given. A new and reliable damage characteristic, spectral area, is proposed for crack location detection.

Secondly, the temperature-sensing characteristics of FBG are analyzed. The temperature characteristics experiment for FBG was carried out to study the effect of temperature on the damage feature spectral area. The experimental results show that the reflection spectra of FBG drifts toward the long wavelength direction as the temperature increases, but the reflection spectra have almost no distortion. The spectral areas display no obvious change with an increase in temperature.

Thirdly, the performance of the spectral area for crack location detection is studied based on simulated and experimental results. The studies show that a significant inflection point appears in the spectral area with crack propagation. The location detection of fatigue crack damage can be performed by identifying the position of the inflection point.

Then, the spectral area is sensitive to the large strain gradient around crack tip along the FBG axis in the fatigue crack propagation experiment. The experimental results show that the spectral area is robust to the experimental noise by comparing the response of the FBG sensors in different positions. In the temperature characteristics experiment, the robustness of spectral area to temperature variations and experimental noise is verified through two parallel experiments.

Finally, the sensitivity of FBG sensors with different grating lengths to crack damage is studied. Compared with the 5-mm FBG sensor, the 10-mm FBG sensors show a larger critical detection range to crack damage.

## Figures and Tables

**Table 1:** The main properties of aluminium alloy 7075-T6.

Material	Tensile Strength Ultimate (MPa)	Tensile Strength Yield (MPa)	Poisson’s Ratio	Elastic Modulus (GPa)
AL7075-T6	560	480	0.33	70

**Table 2:** The parameters of FBG1, FBG2 and FBG3.

FBG Number	Grating Length (mm)	Effective Index	Bragg Wavelength (nm)	Poisson’s Ratio (MPa)	Average Index Change	Measurement Range (με)
FBG1	5.01	1.458	1550	0.17	1.0 × 10^−4^	±5000
FBG2	10.01	1.460	1550	0.17	1.0 × 10^−4^	±5000
FBG3	10.01	1.460	1550	0.17	1.0 × 10^−4^	±5000

**Table 3:** The main parameters of FBG4, FBG5, FBG6 and FBG7.

FBG Number	Grating Length (mm)	Effective Index	Bragg Wavelength (nm)	Poisson’s Ratio (MPa)	Average Index Change
FBG4	5.01	1.458	1550	0.17	1.0 × 10^−4^
FBG5	5.01	1.458	1550	0.17	1.0 × 10^−4^
FBG6	10.01	1.460	1550	0.17	1.0 × 10^−4^
FBG7	10.01	1.460	1550	0.17	1.0 × 10^−4^

## References

[1] Kahandawa G.C., Epaarachchi J., Wang H., Lau K.T. (2012). Use of FBG sensors for SHM in aerospace structures. Photonic Sens..

[2] Lee B. (2003). Review of the present status of optical fiber sensors. Opt. Fiber Technol..

[3] Hill K.O., Meltz G. (1997). Fiber Bragg grating technology fundamentals and overview. J. Lightwave Technol..

[4] Besel M., Breitbarth E. (2016). Advanced analysis of crack tip plastic zone under cyclic loading. Int. J. Fatigue.

[5] Majumder M., Gangopadhyay T.K., Chakraborty A.K., Dasgupta K., Bhattacharya D.K. (2008). Fibre Bragg gratings in structural health monitoring—Present status and applications. Sens. Actuators A Phys..

[6] Feng K., Cui J., Jiang X., Li J., Tan J. Analysis and Simulation Method of the Cantilever FBG Sensors. Proceedings of the Seventh International Symposium on Precision Mechanical Measurements.

[7] Jin B., Zhang W., Zhang M., Ren F., Dai W., Wang Y. (2017). Investigation on characteristic variation of the FBG spectrum with crack propagation in aluminum plate structures. Materials.

[8] Zhang W., Zhang M., Wang X., Zhao Y., Jin B., Dai W. (2019). The Analysis of FBG Central Wavelength Variation with Crack Propagation Based on a Self-Adaptive Multi-Peak Detection Algorithm. Sensors.

[9] James S.W., Dockney M.L., Tatam R.P. (1996). Simultaneous independent temperature and strain measurement using in-fibre Bragg grating sensors. Electron. Lett..

[10] Jones R. (2014). Fatigue crack growth and damage tolerance. Fatigue Fract. Eng. Mater. Struct..

[11] Jin X., Yuan S., Chen J. (2019). On crack propagation monitoring by using reflection spectra of AFBG and UFBG sensors. Sens. Actuators A Phys..

[12] Liu Q., Qiao X., Fu H. (2016). Spectra power and bandwidth of fiber Bragg grating under influence of gradient strain. Photonic Sens..

[13] Bao P., Yuan M., Dong S., Song H., Xue J. (2013). Fiber Bragg grating sensor fatigue crack real-time monitoring based on spectrum cross-correlation analysis. J. Sound Vib..

[14] Mizutani Y., Groves R.M. (2011). Multi-functional measurement using a single FBG sensor. Exp. Mech..

[15] Hu H., Li S., Wang J., Wang Y., Zu L. (2016). FBG-based real-time evaluation of transverse cracking in cross-ply laminates. Compos. Struct..

[16] Takeda S., Yamamoto T., Okabe Y., Takeda N. (2007). Debonding monitoring of composite repair patches using embedded small-diameter FBG sensors. Smart Mater. Struct..

[17] Rito R.L., Crocombe A.D., Ogin S.L. (2017). Health monitoring of composite patch repairs using CFBG sensors: Experimental study and numerical modelling. Compos. Part A Appl. Sci. Manufact..

[18] Pandey V.B., Singh I.V., Mishra B.K., Ahmad S., Rao A.V., Kumar V. (2019). A new framework based on continuum damage mechanics and XFEM for high cycle fatigue crack growth simulations. Eng. Fract. Mech..

[19] Zhao Y., Hu D., Zhang M., Dai W., Zhang W. (2020). In Situ Measurements for Plastic Zone ahead of Crack Tip and Continuous Strain Variation under Cyclic Loading Using Digital Image Correlation Method. Metals.

[20] Pan B., Qian K., Xie H., Asundi A. (2009). Two-dimensional digital image correlation for in-plane displacement and strain measurement: A review. Meas. Sci. Technol..

[21] Ma Z., Chen X. (2019). Fiber Bragg gratings sensors for aircraft wing shape measurement: Recent applications and technical analysis. Sensors.

[22] Othonos A.S., Kalli K. (2000). Fiber Bragg Gratings Fundamentals and Applications in Telecommunications and Sensing. Phys. Today.

[23] Li Z. (2012). Mechanism and Experimental Research on Performance Degeneratio of Fiber Bragg Grating Affected by Temperature. Chin. J. Lasers.

[24] Peters K., Studer M., Botsis J., Iocco A., Limberger H., Salathé R. (2001). Embedded optical fiber Bragg grating sensor in a nonuniform strain field: Measurements and simulations. Exp. Mech..

[25] Bergara A., Dorado J.I., Martin-Meizoso A., Martínez-Esnaola J.M. (2017). Fatigue crack propagation in complex stress fields: Experiments and numerical simulations using the Extended Finite Element Method (XFEM). Int. J. Fatigue.

[26] Bernard P.J., Lindley T.C., Richards C.E. (1976). Mechanisms of Overload Retardation during Fatigue Crack Propagation. Fatigue Crack Growth Under Spectrum Loads.

[27] Blaber J., Adair B., Antoniou A. (2015). Ncorr: Open-source 2D digital image correlation matlab software. Exp. Mech..

